# Frequent Loss of Genome Gap Region in 4p16.3 Subtelomere in Early-Onset Type 2 Diabetes Mellitus

**DOI:** 10.1155/2011/498460

**Published:** 2011-06-20

**Authors:** Hirohito Kudo, Mitsuru Emi, Yasushi Ishigaki, Uiko Tsunoda, Yoshinori Hinokio, Miho Ishii, Hidenori Sato, Tetsuya Yamada, Hideki Katagiri, Yoshitomo Oka

**Affiliations:** ^1^Division of Molecular Metabolism and Diabetes, Tohoku University Graduate School of Medicine, 2-1 Seiryo-machi, Aoba-ku, Sendai, Miyagi 980-8575, Japan; ^2^CNV Laboratory, DNA Chip Research Institute, 1-1-43 Suehiro-cho, Tsurumi-ku Yokohama, Kanagawa 230-0045, Japan; ^3^Department of Metabolic Diseases, Center for Metabolic Diseases, Tohoku University Graduate School of Medicine, 2-1 Seiryo-machi, Aoba-ku, Sendai, Miyagi 980-8575, Japan

## Abstract

A small portion of Type 2 diabetes mellitus (T2DM) is familial, but the majority occurs as sporadic disease. Although causative genes are found in some rare forms, the genetic basis for sporadic T2DM is largely unknown. We searched for a copy number abnormality in 100 early-onset Japanese T2DM patients (onset age <35 years) by whole-genome screening with a copy number variation BeadChip. Within the 1.3-Mb subtelomeric region on chromosome 4p16.3, we found copy number losses in early-onset T2DM (13 of 100 T2DM versus one of 100 controls). This region surrounds a genome gap, which is rich in multiple low copy repeats. Subsequent region-targeted high-density custom-made oligonucleotide microarray experiments verified the copy number losses and delineated structural changes in the 1.3-Mb region. The results suggested that copy number losses of the genes in the deleted region around the genome gap in 4p16.3 may play significant roles in the etiology of T2DM.

## 1. Introduction

Type 2 diabetes mellitus (T2DM) is a common metabolic disease, affecting nearly 300 million individuals worldwide. T2DM affects over 10% of adult individuals over 40 years of age in Japan. The continuous increase in the number of patients is a major public health problem worldwide. Loci for rare monogenic forms of diabetes, such as maturity-onset diabetes of the young [1], mitochondrial diabetes [2, 3], and Wolfram syndrome [4], have been elucidated in a limited proportion of patients. However, the etiology of sporadic T2DM remains largely unknown. Accumulating epidemiological evidence [5–8] suggests that genetic factors play an important role in the susceptibility to sporadic T2DM, in addition to environmental factors such as obesity, aging, and exercise. 

To search for susceptibility gene(s) for sporadic T2DM, genome-wide association studies (GWASs) using single nucleotide polymorphism (SNP) markers have been performed. These GWASs and replication studies have found multiple loci, *TCF7L2 *[9], *KCNQ1* [10, 11], and others [12–18], that are associated with susceptibility to T2DM. However, the overall contribution of these SNPs to sporadic T2DM is relatively low; their odds ratio being in the range of 1.1–1.4 [9, 11]. In addition, these associations have not necessarily been replicated in subsequent studies [12–18].

Copy number variations (CNVs) or structural variations, such as deletion or gain of a genomic region, are increasingly recognized as important interindividual genetic variations across the human genome. CNVs account for more nucleotide variation between two individuals than do SNPs [19–21]. Repetitive, multicopy regions, such as segmental duplications and low copy repeats associated with CNV, are regarded as “rearrangement hotspots,” and CNV regions are predisposed to the generation of deletion/duplication events [22]. Such repeat-rich regions were recently found to show 13-fold enrichment of CNVs over the average genomic coverage in a reference assembly [23]. Therefore, CNVs or structural variations are recognized as significant contributors to human genetic disease and disease susceptibility [24].

In the search for susceptibility gene(s) for T2DM genes, we recruited a panel of 100 early-onset Japanese T2DM patients (onset age <35 years) and 100 controls, and performed CNV analysis in the whole genome using the deCODE-Illumina CNV370K BeadChip which focuses on the CNV-rich region of the human genome, followed by validation and characterization using an Agilent region-targeted high-density custom-made oligonucleotide tiling microarray. We found frequent copy number losses within the 1.3-Mb subtelomeric region in a substantial portion of early-onset Japanese T2DM patients. This region surrounds the genome gap in 4p16.3, which is rich in multiple low copy repeats.

## 2. Materials and Methods

### 2.1. Subjects

We considered that the early onset of T2DM reflects the presence of more genetic factors rather than environmental factors. Therefore, we adopted young-onset diabetic patients as case subjects. We studied 100 unrelated Japanese T2DM patients who developed T2DM before 35 years of age. They were recruited at Tohoku University Hospital and affiliated hospitals and medical clinics. Diabetes was diagnosed using the WHO criteria. Type 1 diabetes mellitus was excluded judged from clinical features and existence of anti-GAD (glutamic acid decarboxylase) antibodies or anti-IA-2 (insulinoma-associated antigen-2) antibodies. Patients with diabetes mellitus due to hepatic disease, pancreatic disease, other endocrinological disease, or mitochondrial DNA mutation, or drug-induced diabetes were excluded, judged from laboratory data and clinical history.

We also studied 100 nondiabetic control subjects, using the following criteria: 60 or more years of age, no prior diagnosis of diabetes mellitus, HbA1c less than 6.4% (where HbA1c (%) was estimated as an NGSP (National Glycohemoglobin Standardization Program) equivalent value (%) calculated by the formula HbA1c (%) = HbA1c (JDS: Japan Diabetes Society value) (%) + 0.4%, considering the relational expression of HbA1c (JDS) (%) measured by the previous Japanese standard substance and measurement methods for HbA1c (NGSP) [25] and no family history of T2DM within third-degree relatives, in order to exclude subjects who were more likely to develop diabetes later.

Clinical features available from 100 early-onset T2DM patients and 100 controls are shown in (see Table S1 in Supplementary Material available online at doi: 10.1155/2011/498460). In addition, clinical features of the 13 early-onset T2DM patients with copy number losses in 4p16.3 are shown separately in Supplementary Table S2 and Supplementary Table S3 in comparison with the rest of 100 early-onset T2DM patients without copy number loss (*n* = 87).

Genetic analysis of human subjects was approved by the ethics committee of Tohoku University Graduate School of Medicine. Appropriate informed consent was obtained from all the subjects examined.

### 2.2. Screening with Whole-Genome CNV BeadChip

We screened the whole genome by CNV analysis using the deCODE-Illumina CNV370K BeadChip (Illumina Infinium system, deCODE genetics, Inc., Iceland), which, in addition to Hap300 SNP marker contents, has CNV probes designed to target the CNV-rich region of the whole genome. The CNV part of the platform consists of probes covering CNV-rich regions of the genome, such as megasatellites (tandem repeats >500 bp), duplicons (region flanked by highly homologous segmental duplication >1 kb), unSNPable regions (>15 kb gaps in HapMap SNP map, and 5–15 kb gaps with >2SNPs with Hardy-Weinberg failure), and CNVs registered in the Database of Genomic Variants. The CNV part of probe content consists of 15,559 CNV segments covering 190 Mb, or 6% of the human genome. The platform has been tested in 4000 Icelandic and HapMap samples.

Data analysis of the deCODE-Illumina CNV chip was carried out using DosageMiner software developed by deCODE genetics, and loss/gain analysis consisted of the following four steps; (1) intensity normalization and GC content correction, (2) removal of batch effects using principal component analysis, (3) calling of clusters using a Gaussian mixture model, and (4) determination of CNV type using graphical constraints. In brief, CNVs were identified when CNV events stood out in the data, as all sample intensities for CNV probes should be increased or decreased relative to neighboring probes that are not in a CNV region. To determine deviations in signal intensity we started by normalizing the intensities. The normalized intensities for each color channel were determined by an equation and fit formula developed by deCODE genetics. A stretch with occurrence of more than one marker showing abnormality in the copy number in a consecutive stretch in the genome is considered more likely to be evidence of deletion or gain [26]. We display Supplementary Table to present raw data, that is, log_2_ ratio measured at each probe for every individual. Raw data at screening step via deCODE/Illumina beads chip for all probes on chromosome 4p is shown in Supplementary Table S4.

### 2.3. High-Density Custom-Made Oligonucleotide Tiling Microarray Analysis

DNA samples from 13 early-onset T2DM patients and 15 control individuals were subjected to Agilent's high-density custom-made oligonucleotide tiling microarray analysis based on an array comparative genomic hybridization (aCGH) assay. We fabricated a custom-designed microarray targeted to a 1.3-Mb genome region in the subtelomere at 4p16.3 (Chr. 4: 550,000-1,850,000 (NCBI Build 36.1, hg18)) according to previously described methods [27, 28]. In brief, we used the Agilent website (http://earray.chem.agilent.com/earray/) to select and design our custom tiling array; the array consisted of probes 60-mer in size (Agilent Technologies, Santa Clara, CA).

Tiling-aCGH experiments were performed essentially as described previously [29]. In brief, test and reference (NA19000, a Japanese male from HapMap project) genomic DNAs (250 ng per sample) were fluorescently labeled with Cy5 (test) and Cy3 (reference) with a ULS Labeling Kit (Agilent Technologies).

 For each sample, respective labeling reactions were mixed and then separated prior to hybridizing to each of the arrays. Labeled test and reference DNAs were combined, denatured, preannealed with Cot-1 DNA (Invitrogen) and blocking agent, and then hybridized to the arrays for 24 hr in a rotating oven at 65°C and 20 rpm (Agilent Technologies). After hybridization and washes, the arrays were scanned at 3 *μ*m resolution with an Agilent G2505C scanner. Images were analyzed with Feature Extraction Software 10.7.3.1 (Agilent Technologies), with the CGH_107_Sep09 protocol for background subtraction and normalization. Detection of abnormal copy number, losses, and gains, in a complex multicopy variable region by high-density tiling array was accessed by deviation of probe log_2_ ratios that exceeded a threshold of 1 SD from the median probe ratio, according to procedures described previously [29–31]. We defined two copy number classes, that is, “unchanged copy number” and “copy number loss.” “Unchanged copy number” was defined when the log_2_ ratio stays within the mean ± 1 SD distribution among the normal population. “Copy number loss” was called when the downward-deviation of log_2_ ratios exceeded a threshold of 1 SD from the median probe ratio. Raw data, that is, log_2 _ratio measured at each probe for every individual obtained by high-density custom-made tilling array analysis is shown in Supplementary Table S5.

## 3. Results

In searching for CNVs associated with early-onset T2DM, we screened the whole genome by CNV analysis using the deCODE-Illumina CNV370K BeadChip in 100 early-onset Japanese T2DM patients and 100 controls. We found four CNVs that fulfilled our screening criteria: (1) reliable CNV (size over 50-Kb, consisting of over 50 consecutive probes), (2) the association being statistically significant by Fisher's exact test which was accompanied by Bonferroni's correction. Four candidate CNV that fulfilled the criteria; that is, CNV on 4p16.3 (*P* = 6.75 × 10^−3^ by Fisher's exact test, *P* < .05 after Bonferroni's correction), CNV on 16p13.3 (*P* = 3.44 × 10^−4^  by Fisher's exact test, *P* < .05 after Bonferroni's correction), CNV on 16q24.3 (*P* = 9.65 × 10^−3^ by Fisher's exact test, *P* < .05 after Bonferroni's correction), and CNV on 19p 13.3 (*P* = 1.61 × 10^−4^ by Fisher's exact test, *P* < .05 after Bonferroni's correction). Of these CNVs listed, the CNV on 4p16.3 was intriguingly found to be located right on the genome gap-177 region on 4p subtelomere. At this candidate CNV on 4p16.3, 13 out of 100 T2DM patients displayed CN-loss around this CNV marker, compared with 1 out of 100 control samples. Because of its unique overlap with genome gap structure, we focused on this CNV region for further analysis.

**Figure 1:**
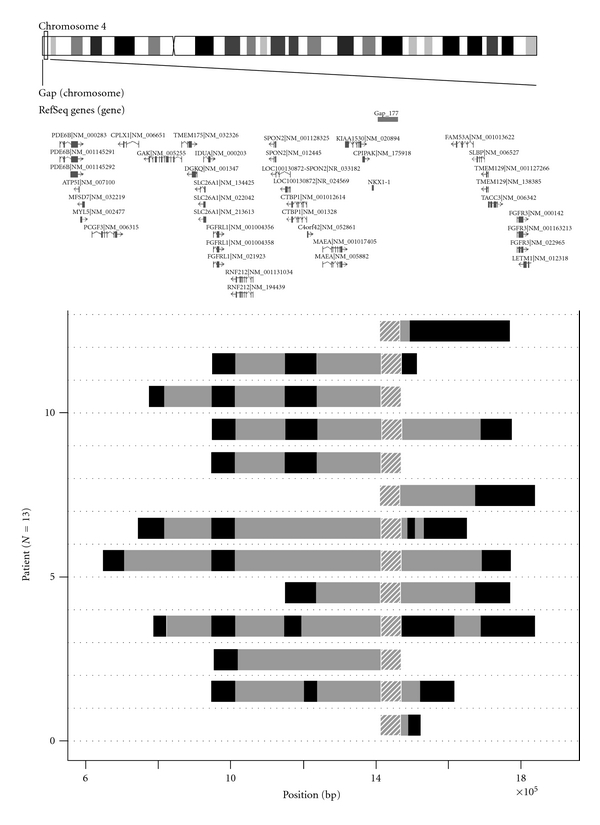
Genomic region harboring copy number loss of 1.3-Mb 4p16.3 subtelomeric region in 13 early-onset T2DM patients. Data measured by deCODE/Illumina CNV370K chip were analyzed by the PennCNV program. Genome structure of the 13 patients are aligned as horizontal bars from genome position 550,000 (left) to position 1,850,000 (right). Hatched region at position 1,423,147–1,478,646 represents genome gap-177 region. Dark solid horizontal bars represent extent of copy number loss in each T2DM patient. Gray regions between the dark bars represent intervals where copy number loss could not be inferred due to poor probe coverage. Upper map shows ideogram of chromosome 4 and the positions of putative genes in 4p16.3 region described in Database of Genomic Variants (http://projects.tcag.ca/variation/). Position is given relative to NCBI Build 35 for the chromosome 4.

Figure 1 shows the pattern of alterations in copy number loss observed among the 100 early-onset Japanese 2DM patients. Thirteen patients displayed copy number losses around the gap in the 4p16.3 subtelomere whereas only one of 100 control samples showed copy number losses in this region. The association was statistically significant by Fisher's exact test (*P* = 6.75 × 10^−3^, OR = 14.7, 95% confidence interval 3.02–72.3). We observed two copy number classes, that is, “unchanged copy number” and “copy number loss” at 1.3-Mb region of chromosome 4p16.3 in our diabetic or control populations. The latter was found frequently observed among early-onset T2DM patients at the 4p16.3 subtelomere. The position, length, or pattern of deletion between one copy number loss in control and 13 copy number losses in T2DM patients were not apparently distinct, although the case number is too small to draw meaningful conclusion.

To verify the CNV BeadChip results, we analyzed copy number changes along the 1.3-Mb region in the subtelomere surrounding the genome gap of 4p16.3 using a high-density custom-made oligonucleotide tiling microarray. We used peripheral blood DNA of the 13 early-onset T2DM patients, identified, and 15 control healthy individuals. Again, we found frequent copy number losses in regions around the genome gap in all the 13 early-onset T2DM patients, whereas none of 15 healthy individuals showed copy number losses.

**Figure 2:**
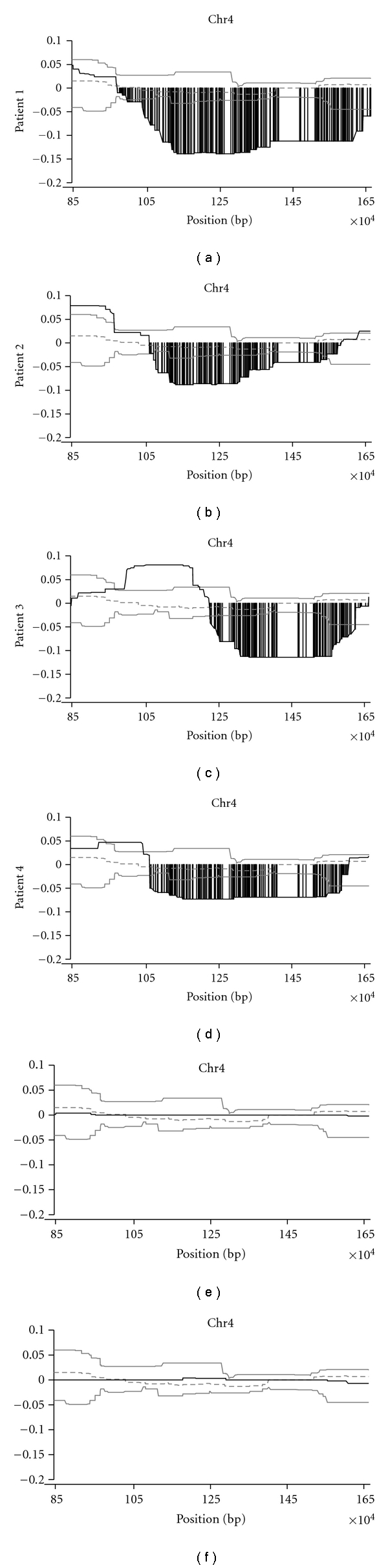
Detailed structure of copy number loss of 1.3-Mb 4p16.3 subtelomeric region resolved by high-density tiling microarray. Log_2_ ratio (*y*-axis) was plotted using moving average along the genome position (*x*-axis). Four representative early-onset T2DM patients are shown as Figures 2(a), 2(b), 2(c), and 2(d), patient 1, 2, 3, and 4, respectively. For comparison, log_2_ ratio of the region of two healthy normal individuals is also displayed as Figures 2(e) and 2(f). Dark line represents copy number plot along the genome. Two light lines indicate normal range of average log_2_ ratios for probes among normal individuals. Dotted line shows median of average log_2_ ratio among normal individuals. Copy number losses are displayed as gray vertical bar. We defined two copy number classes, that is, “unchanged copy number” and “copy number loss.” “Unchanged copy number” was defined when the log_2_ ratio stays within the mean ± 1 SD distribution among the normal population. “Copy number loss” was called when the downward-deviation of log_2_ ratios exceeded a threshold of 1 SD from the median probe ratio.

Figure 2 shows detailed structure of copy number losses in four representative early-onset Japanese T2DM patients with copy number losses, in the 1.3-Mb region in the subtelomere (patients 1, 2, 3, 4; in Figures 2(a), 2(b), 2(c), and 2(d), resp.). Individual copy number plots using moving average (*y*-axis) versus distance along the chromosome (*x*-axis) are shown. As a comparison, copy number plots of healthy individuals who did not exhibit copy number alterations in the region are also shown (Figures 2(e) and 2(f)). 

**Figure 3:**
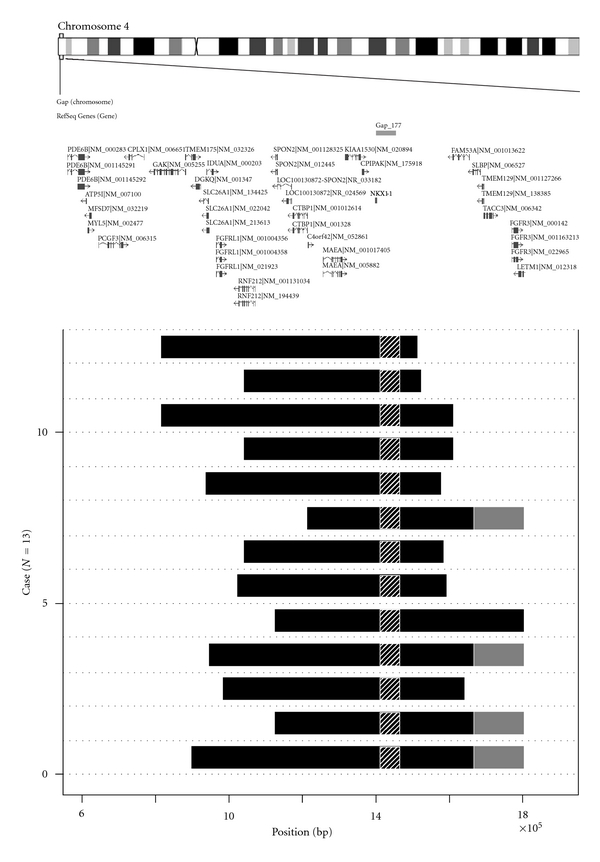
The extent of copy number losses within 1.3-Mb 4p16.3 subtelomeric region in 13 early-onset T2DM patients revealed by high-density oligonucleotide tiling microarray. Dark horizontal bars represent extent of copy number loss region in each patient revealed by Agilent custom tiling array. Genome structure of the 13 patients is aligned as horizontal bars from genome position 550,000 (left) to position 1,850,000 (right). Hatched region at position 1,423,147–1,478,646 represents genome gap-177 region. Gray regions represent proximal ends of stretch where copy number status was not inferred due to presence of multiple low copy repeats. Upper map shows ideogram of chromosome 4 and the positions of putative genes in 4p16.3 region described in Database of Genomic Variants (http://projects.tcag.ca/variation/). Position is given relative to NCBI Build 35 for the chromosome 4.

Figure 3 shows genomic copy number losses in all the 13 early-onset T2DM patients. High-density tiling custom-made microarray showed segmental losses in the subtelomeric region of 4p16.3 in all the 13 patients. Genomic copy number losses in these patients were clustered around a gap region in the 4p16.3 subtelomeric region.

## 4. Discussion

Our initial genome-wide screening with deCODE-Illumina CNV370K BeadChip for association with early-onset T2DM revealed losses in the subtelomeric region of 4p16.3. Subsequent high-density custom-made oligonucleotide tiling microarray verified copy number losses in this region.

 It is worthy to note that most patients with copy number losses were treated with insulin injection. Urine C-peptide reactivity was not significantly different between the two groups, and only few patients underwent glucagon challenge test, thus, the data are too limited to infer the function of insulin secretion. We did not observe significant differences between two groups as to age of onset, body mass index, postprandial plasma glucose levels, or HbA1c levels. Fasting immunoreactive insulin levels were examined in 5 patients with copy number losses and 14 patients without copy number loss. HOMA-R in the former patients was 6.1 ± 6.8 (mean ± SD) and that in the latter patients was 5.5 ± 6.0 (mean ± SD); these values were not significantly different. Incidence of dyslipidemia, hypertension, diabetic retinopathy, nephropathy, or neuropathy was not different between the two groups (Supplementary Tables S2, S3). Further investigation of a large panel of patients would be necessary to clarify any clinical differences that might be present between the two groups.

The current map of CNV in the human genome reported in the existing databases is far from complete [32]. Increasing numbers of CNVs have recently been identified around repetitive sequences such as segmental duplications or low copy repeats. In fact, these repeat-rich regions were found to be 13-fold enriched in CNV over the average genomic coverage in the reference assembly [23]. Probes for conventional genome-wide SNP genotyping platforms are likely to be underrepresented; that is, only 25% and 40% of CNV are covered by the HumanHap300 and HumanHap550 platform, respectively [23]. These recent findings may partly explain why earlier genome-wide association studies for CNVs in the T2DM population failed to detect CNV loci being strongly associated with T2DM [20, 21, 33, 34].

We found copy number losses among early-onset Japanese T2DM patients in a region surrounding a genome gap (gap-177) in the subtelomere of chromosome 4p16.3. The physical map of the human genome still contains a significant number of genome gaps; over 300 gaps still remain in the human draft genome sequence [35] that are considered inaccessible by most existing genotyping and sequencing technologies [36]. These gap regions are estimated to harbor ~1000 genes, which comprise approximately 5% of the human genome (~200 Mb). In particular, they are abundant in subtelomeres and pericentromeric regions of chromosomes [37, 38]. Some of these gaps are thought to be susceptible sites for mediating meiotic recombination and are also susceptibility sites for break points for deletions [39, 40].

Many CNVs were recently identified in repeat-rich regions [41], which are predisposed to the generation of deletion/duplication events [22]. It is intriguing to note that the locus-specific mutation rates for CNV or structural rearrangements were estimated to be between 10^−6^ and 10^−4^: two to four orders of magnitude greater than nucleotide-specific rates for base substitutions or point mutations [19].

The deleted CNV region found in the present study and its flanking region contained 34 genes (Table 1). Gene(s) in this region may predispose to T2DM.

Genes involved in the glucose-induced insulin secretion cascade of pancreatic beta-cells are located in the region, such as ATP5I (ATP synthase, H^+^ transporting, mitochondrial F_0_ complex, subunit E) [42, 43], CPLX1 (complexin 1) [44, 45], GAK (cyclin G associated kinase) in association with CDK5 (cyclin-dependent protein kinases 5) [46–50], and CRIPAK (cysteine-rich PAK1 inhibitor), which is a negative regulator of PAK1 (p21 protein (Cdc42/Rac-) activated kinase 1) [51, 52]. 

FGFR3 (fibroblast growth factor receptor 3) and FGFRL1 (fibroblast growth factor receptor-like 1) are suggested to be involved in pancreatic development and differentiation [53–56]. TACC3 (transforming, acidic coiled-coil containing protein 3) may be involved in embryonic development including the pancreas [57, 58]. 

CTBP1 (C-terminal binding protein 1) and MAEA (macrophage erythroblast attacher) have effects on adipose tissue functions [59, 60], which may lead to insulin resistance. NKX1-1(NK1 homeobox 1) may cause insulin resistance with a function in the maintenance of energy homeostasis [61].

Recently, through whole-genome screening of a copy number variation using a CNV BeadChip and real-time quantitative polymerase chain reaction (qPCR), Kato et al. identified a segmental copy number gain within the 40-kb region on 10p15.3 subtelomere in patients of sporadic amyotrophic lateral sclerosis (SALS) [62]. They demonstrated the copy number gain in 46 out of 83 SALS patients, as compared with 10 out of 99 controls. The copy number gain region they identified is rather small (40-kb) and harbored two genes encoding isopentenyl diphosphate isomerase 1 (IDI1) and IDI2. Thus, they suggested the copy number gain in the region of these genes may play a significant role in the pathogenesis of SALS. The present study share a similar genome abnormality in that we found frequent copy number alterations in subtelomere region among sporadic adult-onset disease of unknown cause. The copy number alterations we identified here in early-onset T2DM were copy number losses on different chromosome subtelomere (4p16.3) and the size is rather large (up to 1.3-Mb). In our case, we suspect that multiple genes in the region may be involved in diabetes pathogenesis through impairments caused by copy number losses.

## 5. Conclusion

These results suggested that copy number losses of the candidate genes in the deleted region surrounding the genome gap in 4p16.3 may play significant roles in the etiology of T2DM. Further functional study, as well as investigation of 4p16.3 loss in a large panel of early-onset T2DM patients in different ethnic populations and geographical regions, is warranted.

## Figures and Tables

**Table 1:** Putative genes located within 1.3-Mb region in 4p16.3 subtelomere.

Start	End	Cytogenetic location	Symbol	Description	Model evidence
609,373	654,571	4p16.3	PDE6B	Phosphodiesterase 6B, cGMP-specific, rod, beta (congenital stationary night blindness 3, autosomal dominant)	Best RefSeq
656,225	658,122	4p16.3	ATP5I	ATP synthase, H+ transporting, mitochondrial F0 complex, subunit E	Best RefSeq
661,711	665,817	4p16.3	MYL5	Myosin, light chain 5, regulatory	Best RefSeq
665,618	672,973	4p16.3	MFSD7	Major facilitator superfamily domain containing 7	mRNA
689,573	754,428	4p16.3	PCGF3	Polycomb group ring finger 3	Best RefSeq
719,829	721,544	4p16.3	LOC100128084	Hypothetical protein LOC100128084	mRNA
764,588	765,632	4p16.3	LOC100129917	Hypothetical protein LOC100129917	mRNA
768,745	809,945	4p16.3	CPLX1	Complexin 1	mRNA
833,065	916,174	4p16.3	GAK	Cyclin G associated kinase	mRNA
916,262	942,444	4p16.3	TMEM175	Transmembrane protein 175	Best RefSeq
942,675	957,344	4p16.3	DGKQ	Diacylglycerol kinase, theta 110 kDa	Best RefSeq
962,861	977,224	4p16.3	SLC26A1	Solute carrier family 26 (sulfate transporter), member 1	Best RefSeq
970,785	988,317	4p16.3	IDUA	Iduronidase, alpha-L-	mRNA
995,610	1,010,686	4p16.3	FGFRL1	Fibroblast growth factor receptor-like 1	Best RefSeq
1,044,654	1,045,386	4p16.3	LOC100132787	Hypothetical protein LOC100132787	mRNA
1,055,269	1,097,350	4p16.3	RNF212	Ring finger protein 212	Best RefSeq
1,116,050	1,129,814	4p16.3	LOC100133135	Hypothetical protein LOC100133135	Protein
1,135,541	1,135,957	4p16.3	FLJ35816	FLJ35816 protein	Protein
1,149,293	1,149,712	4p16.3	LOC100131106	Hypothetical protein LOC100131106	Protein
1,150,723	1,156,597	4p16.3	SPON2	Spondin 2, extracellular matrix protein	Best RefSeq
1,184,431	1,185,198	4p16.3	LOC100130872	Hypothetical protein LOC100130872	mRNA
1,195,228	1,232,908	4p16.3	CTBP1	C-terminal binding protein 1	Best RefSeq
1,234,177	1,236,616	4p16.3	C4orf42	Chromosome 4 open reading frame 42	Best RefSeq
1,273,672	1,323,925	4p16.3	MAEA	Macrophage erythroblast attacher	Best RefSeq
1,331,104	1,371,732	4p16.3	KIAA1530	KIAA1530	mRNA
1,375,340	1,379,782	4p16.3	CRIPAK	Cysteine-rich PAK1 inhibitor	mRNA
1,380,072	1,392,453	4p16.3	NKX1-1	NK1 homeobox 1	Protein
1,484,196	1,519,086	4p16.3	LOC100133199	Similar to RE32881p	mRNA
1,611,605	1,655,516	4p16.3	FAM53A	Family with sequence similarity 53, member A	Best RefSeq
1,664,325	1,683,828	4p16.3	SLBP	Stem-loop (histone) binding protein	Best RefSeq
1,687,477	1,692,882	4p16.3	TMEM129	Transmembrane protein 129	Best RefSeq
1,693,062	1,716,696	4p16.3	TACC3	Transforming, acidic coiled-coil containing protein 3	Best RefSeq
1,765,421	1,780,396	4p16.3	FGFR3	Fibroblast growth factor receptor 3 (achondroplasia, thanatophoric dwarfism)	Best RefSeq
1,784,558	1,827,772	4p16.3	LETM1	Leucine zipper-EF-hand containing transmembrane protein 1	Best RefSeq

## References

[1] Frayling TM, Evans JC, Bulman MP (2001). beta-cell genes and diabetes: molecular and clinical characterization of mutations in transcription factors. *Diabetes*.

[2] van den Ouweland JM, Lemkes HH, Ruitenbeek W (1992). Mutation in mitochondrial tRNA(Leu)(UUR) gene in a large pedigree with maternally transmitted type II diabetes mellitus and deafness. *Nature Genetics*.

[3] Oka Y, Katagiri H, Yazaki Y, Murase T, Kobayashi T (1993). Mitochondrial gene mutation in islet-cell-antibody-positive patients who were initially non-insulin-dependent diabetics. *The Lancet*.

[4] Inoue H, Tanizawa Y, Wasson J (1998). A gene encoding a transmembrane protein is mutated in patients with diabetes mellitus and optic atrophy (Wolfram syndrome). *Nature Genetics*.

[5] Kaprio J, Tuomilehto J, Koskenvuo M (1992). Concordance for Type 1 (insulin-dependent) and Type 2 (non-insulin-dependent) diabetes mellitus in a population-based cohort of twins in Finland. *Diabetologia*.

[6] Poulsen P, Kyvik KO, Vaag A, Beck-Nielsen H (1999). Heritability of type II (non-insulin-dependent) diabetes mellitus and abnormal glucose tolerance—a population-based twin study. *Diabetologia*.

[7] Meigs JB, Cupples LA, Wilson PW (2000). Parental transmission of type 2 diabetes: the Framingham Offspring Study. *Diabetes*.

[8] Weijnen CF, Rich SS, Meigs JB, Krolewski AS, Warram JH (2002). Risk of diabetes in siblings of index cases with Type 2 diabetes: implications for genetic studies. *Diabetic Medicine*.

[9] Grant SF, Thorleifsson G, Reynisdottir I (2006). Variant of transcription factor 7-like 2 (TCF7L2) gene confers risk of type 2 diabetes. *Nature Genetics*.

[10] Unoki H, Takahashi A, Kawaguchi T (2008). SNPs in KCNQ1 are associated with susceptibility to type 2 diabetes in East Asian and European populations. *Nature Genetics*.

[11] Yasuda K, Miyake K, Horikawa Y (2008). Variants in KCNQ1 are associated with susceptibility to type 2 diabetes mellitus. *Nature Genetics*.

[12] Saxena R, Voight BF, Lyssenko V (2007). Genome-wide association analysis identifies loci for type 2 diabetes and triglyceride levels. *Science*.

[13] Scott LJ, Mohlke KL, Bonnycastle LL (2007). A genome-wide association study of type 2 diabetes in Finns detects multiple susceptibility variants. *Science*.

[14] Sladek R, Rocheleau G, Rung J (2007). A genome-wide association study identifies novel risk loci for type 2 diabetes. *Nature*.

[15] Steinthorsdottir V, Thorleifsson G, Reynisdottir I (2007). A variant in CDKAL1 influences insulin response and risk of type 2 diabetes. *Nature Genetics*.

[16] Zeggini E, Weedon MN, Lindgren CM (2007). Replication of genome-wide association signals in UK samples reveals risk loci for type 2 diabetes. *Science*.

[17] Prokopenko I, McCarthy MI, Lindgren CM (2008). Type 2 diabetes: new genes, new understanding. *Trends in Genetics*.

[18] Zeggini E, Scott LJ, Saxena R (2008). Meta-analysis of genome-wide association data and large-scale replication identifies additional susceptibility loci for type 2 diabetes. *Nature Genetics*.

[19] Lupski JR (2007). Genomic rearrangements and sporadic disease. *Nature Genetics*.

[20] Conrad DF, Pinto D, Redon R (2010). Origins and functional impact of copy number variation in the human genome. *Nature*.

[21] Park H, Kim JI, Ju YS (2010). Discovery of common Asian copy number variants using integrated high-resolution array CGH and massively parallel DNA sequencing. *Nature Genetics*.

[22] Sharp AJ, Locke DP, McGrath SD (2005). Segmental duplications and copy-number variation in the human genome. *American Journal of Human Genetics*.

[23] Cooper GM, Zerr T, Kidd JM, Eichler EE, Nickerson DA (2008). Systematic assessment of copy number variant detection via genome-wide SNP genotyping. *Nature Genetics*.

[24] Redon R, Ishikawa S, Fitch KR (2006). Global variation in copy number in the human genome. *Nature*.

[25] Seino Y, Nanjo K, Tajima N (2010). Report of the committee on the classification and diagnostic criteria of diabetes mellitus. *Journal of the Japan Diabetes Society*.

[26] Stefansson H, Rujescu D, Cichon S (2008). Large recurrent microdeletions associated with schizophrenia. *Nature*.

[27] Barrett MT, Scheffer A, Ben-Dor A (2004). Comparative genomic hybridization using oligonucleotide microarrays and total genomic DNA. *Proceedings of the National Academy of Sciences of the United States of America*.

[28] Perry GH, Ben-Dor A, Tsalenko A (2008). The fine-scale and complex architecture of human copy-number variation. *American Journal of Human Genetics*.

[29] de Smith AJ, Tsalenko A, Sampas N (2007). Array CGH analysis of copy number variation identifies 1284 new genes variant in healthy white males: implications for association studies of complex diseases. *Human Molecular Genetics*.

[30] Sharp AJ, Hansen S, Selzer RR (2006). Discovery of previously unidentified genomic disorders from the duplication architecture of the human genome. *Nature Genetics*.

[31] Lupski JR (2009). Genomic disorders ten years on. *Genome Medicine*.

[32] Ionita-Laza I, Rogers AJ, Lange C, Raby BA, Lee C (2009). Genetic association analysis of copy-number variation (CNV) in human disease pathogenesis. *Genomics*.

[33] Shtir C, Pique-Regi R, Siegmund K, Morrison J, Schumacher F, Marjoram P (2009). Copy number variation in the Framingham Heart Study. *BioMed Central Proceedings*.

[34] The Wellcome Trust Case Control Consortium (2010). Genome-wide association study of CNVs in 16,000 cases of eight common diseases and 3,000 shared controls. *Nature*.

[35] International Human Genome Sequencing Consortium (2004). Finishing the euchromatic sequence of the human genome. *Nature*.

[36] Eichler EE, Flint J, Gibson G (2010). Missing heritability and strategies for finding the underlying causes of complex disease. *Nature Reviews Genetics*.

[37] Lese CM, Fantes JA, Riethman HC, Ledbetter DH (1999). Characterization of physical gap sizes at human telomeres. *Genome Research*.

[38] Eichler EE, Clark RA, She X (2004). An assessment of the sequence gaps: unfinished business in a finished human genome. *Nature Reviews Genetics*.

[39] Devriendt K, Vermeesch JR (2004). Chromosomal phenotypes and submicroscopic abnormalities. *Human genomics*.

[40] Van Buggenhout G, Melotte C, Dutta B (2004). Mild Wolf-Hirschhorn syndrome: micro-array CGH analysis of atypical 4p16.3 deletions enables refinement of the genotype-phenotype map. *Journal of Medical Genetics*.

[41] Itsara A, Cooper GM, Baker C (2009). Population analysis of large copy number variants and hotspots of human genetic disease. *American Journal of Human Genetics*.

[42] Swartz DA, Park EI, Visek WJ, Kaput J (1996). The e subunit gene of murine FF-ATP synthase. Genomic sequence, chromosomal mapping, and diet regulation. *Journal of Biological Chemistry*.

[43] Hayakawa T, Noda M, Yasuda K (1998). Ethidium bromide-induced inhibition of mitochondrial gene transcription suppresses glucose-stimulated insulin release in the mouse pancreatic beta-cell cell line betaHC9. *Journal of Biological Chemistry*.

[44] Abderrahmani A, Niederhauser G, Plaisance V (2004). Complexin I regulates glucose-induced secretion in pancreatic beta-cells. *Journal of Cell Science*.

[45] Dubois M, Vacher P, Roger B (2007). Glucotoxicity inhibits late steps of insulin exocytosis. *Endocrinology*.

[46] Kanaoka Y, Kimura SH, Okazaki I, Ikeda M, Nojima H (1997). GAK: a cyclin G associated kinase contains a tensin/auxilin-like domain. *Federation of European Biochemical Societies Letters*.

[47] Kimura SH, Tsuruga H, Yabuta N, Endo Y, Nojima H (1997). Structure, expression, and chromosomal localization of human GAK. *Genomics*.

[48] Wei FY, Nagashima K, Ohshima T (2005). Cdk5-dependent regulation of glucose-stimulated insulin secretion. *Nature Medicine*.

[49] Ubeda M, Rukstalis JM, Habener JF (2006). Inhibition of cyclin-dependent kinase 5 activity protects pancreatic beta cells from glucotoxicity. *Journal of Biological Chemistry*.

[50] Kitani K, Oguma S, Nishiki T (2007). A Cdk5 inhibitor enhances the induction of insulin secretion by exendin-4 both in vitro and in vivo. *Journal of Physiological Sciences*.

[51] Talukder AH, Meng Q, Kumar R (2006). CRIPak, a novel endogenous Pak1 inhibitor. *Oncogene*.

[52] Wang Z, Oh E, Thurmond DC (2007). Glucose-stimulated Cdc42 signaling is essential for the second phase of insulin secretion. *Journal of Biological Chemistry*.

[53] Wiedemann M, Trueb B (2000). Characterization of a novel protein (FGFRL1) from human cartilage related to FGF receptors. *Genomics*.

[54] Hardikar AA, Marcus-Samuels B, Geras-Raaka E, Raaka BM, Gershengorn MC (2003). Human pancreatic precursor cells secrete FGF2 to stimulate clustering into hormone-expressing islet-like cell aggregates. *Proceedings of the National Academy of Sciences of the United States of America*.

[55] Arnaud-Dabernat S, Kritzik M, Kayali AG (2007). FGFR3 is a negative regulator of the expansion of pancreatic epithelial cells. *Diabetes*.

[56] Gerber SD, Steinberg F, Beyeler M, Villiger PM, Trueb B (2009). The murine Fgfrl1 receptor is essential for the development of the metanephric kidney. *Developmental Biology*.

[57] Sadek CM, Jalaguier S, Feeney EP (2000). Isolation and characterization of AINT: a novel ARNT interacting protein expressed during murine embryonic development. *Mechanisms of Development*.

[58] Sadek CM, Pelto-Huikko M, Tujague M, Steffensen KR, Wennerholm M, Gustafsson JA (2003). TACC3 expression is tightly regulated during early differentiation. *Gene Expression Patterns*.

[59] Qiu J, Ni YH, Chen RH (2008). Gene expression profiles of adipose tissue of obese rats after central administration of neuropeptide Y-Y5 receptor antisense oligodeoxynucleotides by cDNA microarrays. *Peptides*.

[60] Vernochet C, Peres SB, Davis KE (2009). C/EBPalpha and the corepressors CtBP1 and CtBP2 regulate repression of select visceral white adipose genes during induction of the brown phenotype in white adipocytes by peroxisome proliferator-activated receptor gamma agonists. *Molecular and Cellular Biology*.

[61] Simon R, Lufkin T, Bergemann AD (2007). Homeobox gene Sax2 deficiency causes an imbalance in energy homeostasis. *Developmental Dynamics*.

[62] Kato T, Emi M, Sato H (2010). Segmental copy-number gain within the region of isopentenyl diphosphate isomerase genes in sporadic amyotrophic lateral sclerosis. *Biochemical and Biophysical Research Communications*.

